# Cohort Profile: evaluation of the targeted individual promotion in german preschools using the revised Dortmund Developmental Screening for Preschools DESK 3–6 R (project “GIF MV”)

**DOI:** 10.1186/s12889-023-15307-5

**Published:** 2023-03-01

**Authors:** Vanessa Sophie Ernst, Marco Franze, Anika Kästner, Wolfgang Hoffmann

**Affiliations:** Institute for Community Medicine, Section Epidemiology of Health Care and Community Health, Research Unit Prevention, University Medicine Greifswald, Ellernholzstr. 1-2, 17487 Greifswald, Germany

**Keywords:** Early prevention, Preschools, Developmental risks, Developmental screening, Social inequalities

## Abstract

This dynamic cohort was established to evaluate the targeted individual promotion of children affected by developmental risks as part of the German federal state law for child day-care and preschools in Mecklenburg-Western Pomerania. The project has been conducted in preschools in regions with a low socio-economic profile since 2011. Since 2017, the revision of the standardized Dortmund Developmental Screening for Preschools (DESK 3–6 R) has been applied. Developmental risks of 3 to 6-year-old children in the domains of motor, linguistic, cognitive and social competencies are monitored. The cohort is followed up annually. In 2020, n = 7,678 children from n = 152 preschools participated. At the baseline (2017), n = 8,439 children participated. Due to the defined age range of this screening, 3,000 to 4,000 5-6-year-old children leave the cohort annually. Simultaneously, an approximately equal number of 3-year-old children enters the cohort per survey wave. N = 702 children participated in all 4 survey waves. On the basis of DESK 3–6 R scores available from survey waves 2017 to 2019 it is possible to compute expected values for the survey wave 2020 and to compare those with the measured values to evaluate the effects of the COVID-19 pandemic (i.e. parental home care due to restrictions related to COVID-19).

## Background

Socioeconomic conditions influence the health and development of children. This influence starts in the womb and continues well into adulthood. Data from the KiGGS Study show for example that children from lower socioeconomic backgrounds are more likely to have an increased risk for psychological problems than children from an intermediate or high socioeconomic background [1]. The KiGGS Study also revealed that the prevalence of ADHD diagnoses as reported by parents is nearly twice as high among children from a low socioeconomic background compared to children from families with an intermediate or high socioeconomic status [2]. In both instances the association remains over time. Another difference between children of different socioeconomic backgrounds is found with regard to exercise and participation in sports clubs. Children from families with a low socioeconomic status exercise less and have a lower participation rate in sports clubs than children from higher socioeconomic backgrounds [3]. Exercise is important to foster motor abilities in children, while participation in sports clubs additionally helps to develop social abilities. As children from families with low socioeconomic status are disadvantaged in both these areas this could negatively impact their development compared to peers from families with higher socioeconomic status.

According to the German Index of Socioeconomic Deprivation the German federal state Mecklenburg-Western Pomerania (MWP) is one of the most socioeconomic deprived regions of Germany [4]. MWP is characterized by a lower household income (2020: MWP 21,396€ vs. Germany 28,610€ [5]) and a higher unemployment rate than the German average (unemployment rate in January 2023: MWP 8.3% vs. Germany 5.7% [6]).

The school entry examinations in MWP show that a substantial proportion of children is affected by developmental delays. In the school year 2016/17, the school entry examinations revealed that up to 22% of the children had developmental delays in language, 14.7% in gross motor, 15.3% in fine motor development, and 15.6% had limited psychophysical capacity [7]. These children start school under unfavourable conditions. To counter these risks, the early detection of developmental risks in children in preschools with a low socioeconomic profile was made part of the federal state law for child day-care and preschools in MWP, which was enacted in 2011 (Kindertagesförderungsgesetz; KiföG M-V) [8, 9].

According to the KiföG M-V preschools in MWP have the opportunity to register for additional funding if an above average proportion of parental fees are covered by the youth welfare office (Jugendamt). The conditions which need to be fulfilled to receive this funding include an annual validated developmental screening to detect developmental risks (DESK 3–6 R), the subsequent targeted individual promotion of children at risk, and participation in a scientific evaluation (project GIF M-V) [10, 11]. In the following sections of this paper the preschools participating in this programme are referred to as “DESK preschools”.

GIF M-V is conducted by the Institute for Community Medicine, Section Epidemiology of Health Care and Community Health (ICM-VC) which is part of the University Medicine Greifswald. This project is funded by the Ministry of Education and Daycare Facilities for Children MWP. Its aim is to evaluate the effectiveness of the targeted individual promotion in DESK preschools.

## Methods

### Participants

**Fig. 1:**
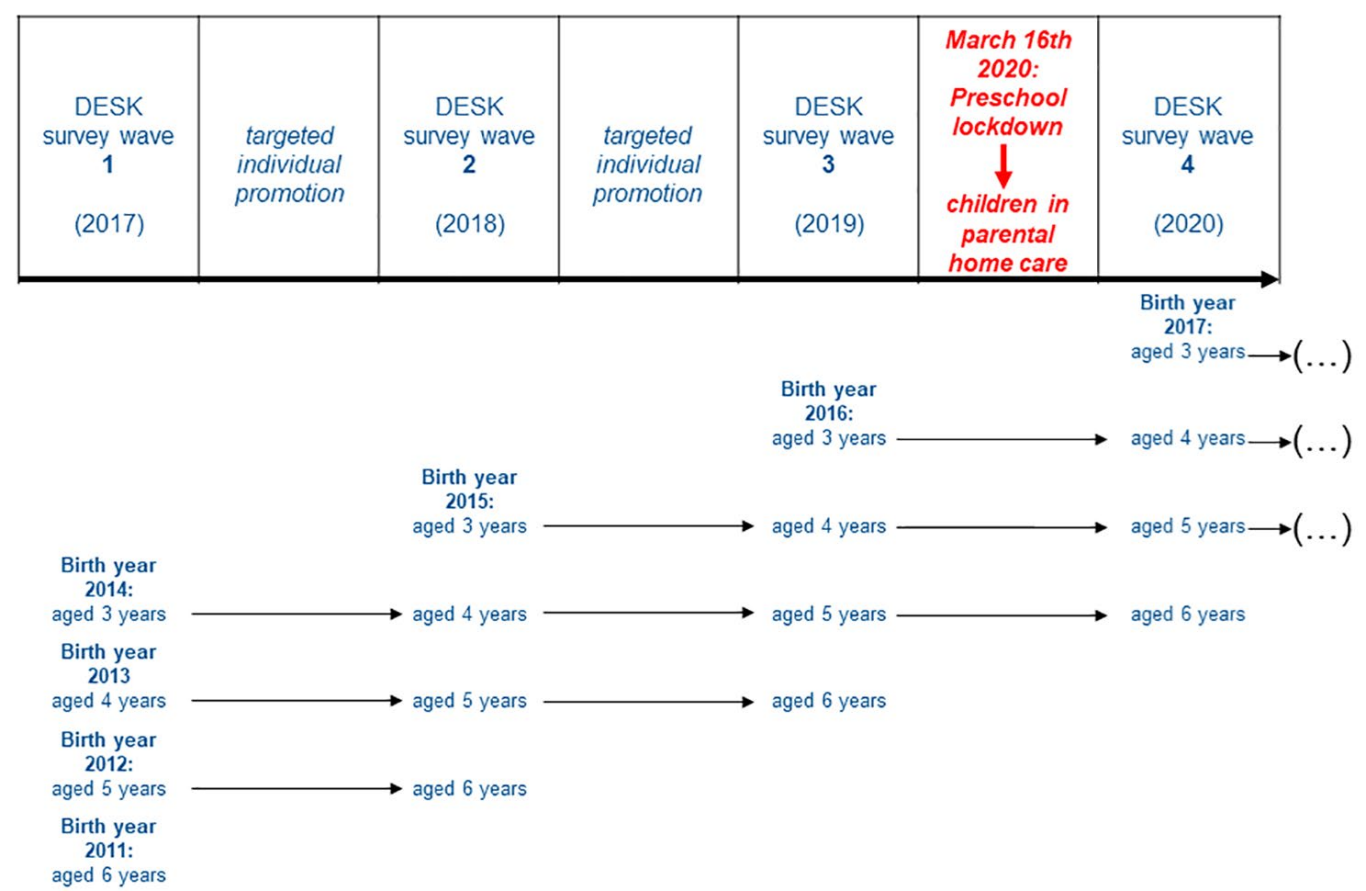
Overview of baseline and follow-up for DESK 3–6 R over time

In this dynamic prospective cohort, the participants are children aged 3–6 years attending a DESK preschool (Table 1). Due to this design, it is possible to perform a longitudinal assessment of the developmental risks of each individual child over up to four consecutive survey waves (SW) (e.g. see birth year 2014 in Fig. 1).

Participation in the screening is mandatory for all children in the DESK preschool but the written consent of parents or legal guardians is required before data from the DESK 3–6 R questionnaires can be forwarded from the preschool to the ICM-VC. For this reason, parents or legal guardians are informed in advance by the preschool teachers, receive an information sheet about the evaluation and usage of data, and provide written informed consent. Because of the dynamic character of this prospective cohort study the number of children may vary which is why response rate is rather an approximation (a reason for this is that children may leave or start attending a DESK preschool during the survey wave making it difficult to report the total number of children in a DESK preschool). However, over the past years less than 5% of parents have refused permission for passing completed DESK questionnaires on to the ICM-VC [12]. Missing data has to be accepted due to data protection issues (i.e. the freedom of parents to refuse transmitting data to the ICM-VC).

Preschools are selected for the program for a minimum of three years. Therefore, the follow-up is conducted annually until the children

1. start elementary school,
2. leave the preschool because of moving house, or
3. the preschool no longer qualifies for the additional funding.

The DESK 3–6 R is conducted annually starting in May till the end of November by preschool teachers. The preschool teachers are trained by experts from the ICM-VC before conducting the examination. Furthermore, teachers have the possibility to refresh their knowledge and skills by participating in the training again.

**Table 1:** Number of participants per survey wave by sex and age

	SW1 (2017)		SW 2 (2018)		SW 3 (2019)		SW 4 (2020)	
**N**	8,347^1^		7,678^1^		7,890^1^		7,547^1^	
	**female**	**male**	**female**	**male**	**female**	**male**	**female**	**male**
**3-year olds**	1,078	1,010	985	1,002	965	1,001	898	851
**4-year olds**	1,178	1,206	1,116	1,168	1,137	1,136	1,069	1,092
**5-year olds**	1,288	1,355	1,124	1,213	1,186	1,266	1,163	1,216
**6-year olds**	579	653	521	849	592	607	605	653
**Total**	4,123	4,224	3,746	3,932	3,880	4,010	3,735	3,812

^1^Not included: Missing values for age and sex (SW 1 n = 92; SW 2 n = 57; SW 3 n = 57; SW 4 n = 131)

Between 2011 and 2016 the examination was conducted with the original version of the Dortmund Developmental Screening for Preschools (Dortmunder Entwicklungsscreening für den Kindergarten; DESK 3–6). In 2016 the instrument was revised leading to the Dortmund Developmental Screening for Preschools – Revision (Dortmunder Entwicklungsscreening für den Kindergarten – Revision; DESK 3–6 R) [11]. The revised version of the screening has been used in the project since 2017 (Table 2).

**Table 2:** Overview of measuring tools in each time period

Phase	Annual measurements
2011–2016	DESK 3–6
2014–2016	Questionnaire for preschool directors
2017 to date	DESK 3–6 R
2018; 2020 to date	Questionnaire for preschool directors

Since the first survey wave in 2017 (SW 1, “baseline”) three more waves using the DESK 3–6 R have been conducted, namely in 2018 (SW2), 2019 (SW3) and 2020 (SW4), thus there have been four waves in total so far (Fig. 1). Data from 2021 (SW 5) and 2022 (SW6) are currently being processed. A seventh wave is going to be conducted in 2023.

**Fig. 2:**
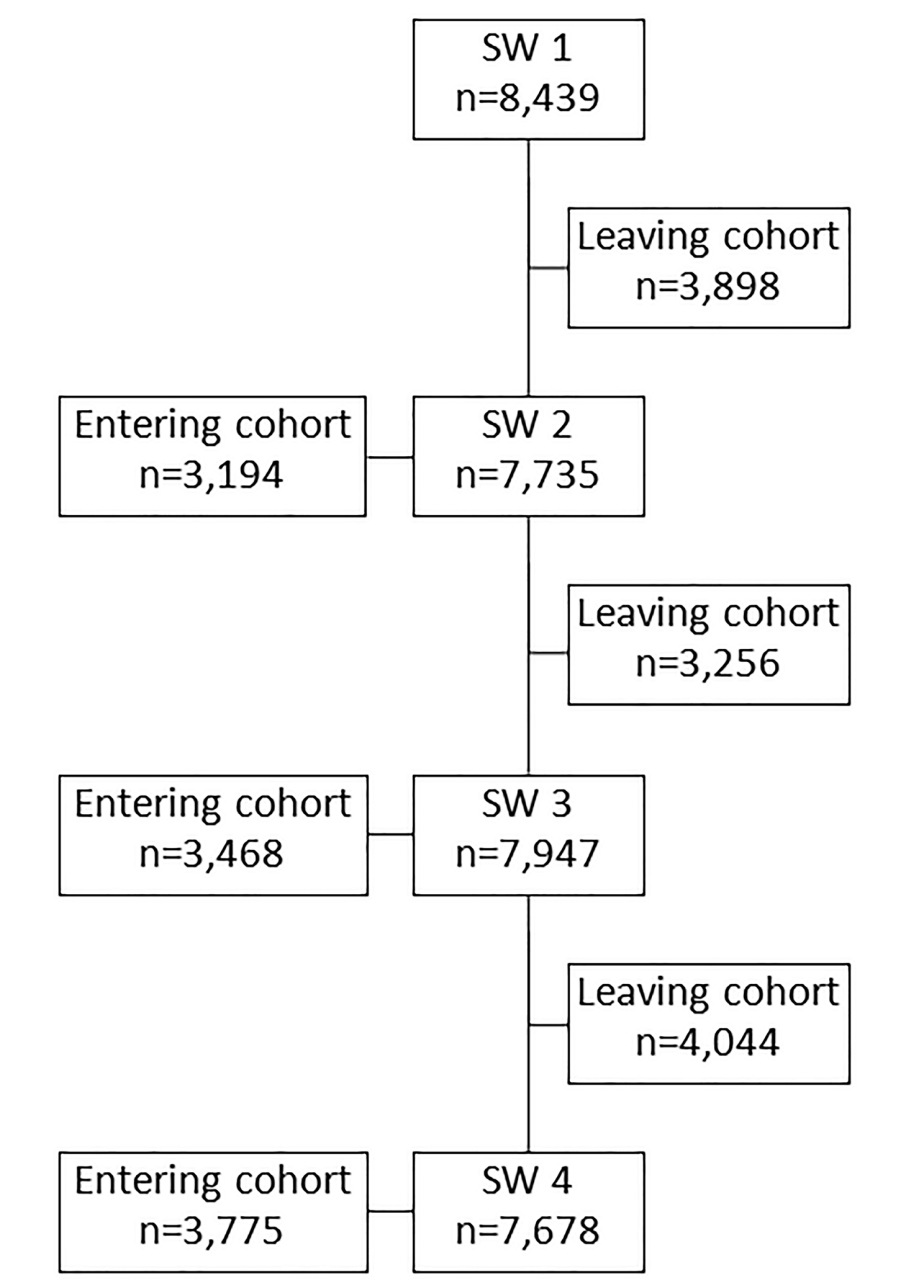
Loss and entry of new cases for DESK 3–6 R per SW

On average, 8,000 children participated in each survey wave. Due to the defined age range of the DESK 3–6 R, about 3,000 to 4,000 children dropped out of the cohort between each wave (Fig. 2). This was balanced out by a largely comparable number of young children entering the cohort. A total of n = 702 children participated in all four survey waves.

The number of participating preschools also varied between waves. In SW1 n = 161 preschools, in SW2 n = 154 preschools and in SW3 and SW4 n = 152 preschools participated.

The composition of cases in the follow-up measurements largely follows a deterministic pattern: New children entering the cohort switch from nursery schools (ages 0–2) to preschools (ages 3–6) when they turn 3 years old. Most 6-year olds drop out of the cohort after the survey wave because they start elementary school and thus no longer attend a preschool. Nevertheless, children enter, change or leave preschools for various reasons at other points in time. For instance, children may start preschool at a different age after having been cared for at home or in some other arrangement. Based on the collected data, these processes follow a random pattern. Furthermore, preschools may no longer qualify for the program at a certain point and are therefore no longer followed up. It is however possible that these preschools later re-enter the program, which allows the measurement of developmental risks to continue with the gaps in the data restricted to single survey waves. Most new cases stem from new DESK preschools being accepted into the program.

To comply with data privacy requirements only pseudonymized data can be used. Personal data (i.e. first name, last name, birth date, sex) are only used to generate the pseudonyms and get deleted afterwards. In the case of incomplete data (e.g. a missing birth date, a missing first name, or if the date the DESK was conducted is missing etc.) telephone/email queries are necessary to complete the data. Even only slight differences in the longitudinally assessed personal data can result in different pseudonyms for the same case (e.g. two data entries including both an identical last name, birth date and sex but a slightly different first name: “Marie-Luise” (data entry in survey wave 1) vs. “Marie Luise” (data entry in survey wave 2)). This may lead to a false-negative record-linkage (i.e. when the two data entries are falsely considered to belong to two different children). To minimize this source of error, the ID Management solution Enterprise Identifier Cross Referencing (E-PIX) is used [13, 14]. The goal of E-PIX is to avoid duplicate participant entries by applying the Fellegi-Sunter algorithm and the Levenshtein distance [15]. The independent software module enables unambiguous participant management and efficient aggregation of research data and supports the correction of potential synonym errors (false-negative record linkage) over the course of data assessment. E-PIX and all further MOSAIC tools are made available under open source licensing on a project portal (mosaic-greifswald.de; MOSAIC is funded by the German Research Foundation DFG; grant number HO 1937/2 − 1; [13, 14]).

### Measures

Developmental risks are assessed based on the DESK 3–6 R. The DESK 3–6 R is a standardised, validated, objective and reliable tool to assess early signs of developmental risks [11, 16]. The tool is not used to diagnose clinically relevant developmental delays as the preschool teachers are not trained for this. Instead, it is used as an early alarm system to trigger preventive action to stop a further decline in competencies. It also generates a signal for parents to seek out professional help outside the preschool, for example advice from a paediatrician. In comparison to similar screening tools tested by preschool teachers DESK 3–6 R proved to be the most comprehensive and practical tool to use in preschools [17]. The DESK 3–6 R captures eleven different developmental domains, which are presented in Table 3.

**Table 3:** Domains assessed in DESK 3–6 R according to age group

3 years old	4 years old	5 years old	6 years old
fine motor			
gross motor			
cognition and language	cognition	basic competence mathematics	
		attention and concentration	
	language and communication	language and communication	
		basic competence written language	
social behaviour		social competence	
		social interaction	

As the children get older the domains get more differentiated. This means that not all competencies can be evaluated longitudinally over all age groups. The DESK 3–6 R measures the domains via three types of tasks: (1) tasks implemented in a specific form of group play (“circus game”), (2) individual tasks, and (3) tasks the preschool teachers observe in day-to-day preschool life. The group play allows assessing up to five children at a time but needs to be conducted during individual sessions with the selected children. Individual task can easily be implemented in day-to-day activities without separating single children from the group. Observation tasks rely solely on the observations of the preschool teacher in day-to-day activities with the children. As an example, the following are tasks from the domain attention and concentration:

- “Sets aside his/her own needs within the group.”
- “Waits for his/her turn.”
- “Occupies him- or herself with a task over a longer period of time.”
- “Continues performing an activity even if he or she gets distracted.”
- “Listens carefully to the preschool teacher’s explanations.”
- “Remains seated while eating, playing or doing handicrafts.”
- “Remembers agreements.”
- “Is aware of his or her own belongings.”

The screening is available in 3 different age versions: one for 3-year olds, one for 4-year olds and one for 5- to 6-year olds. There are between eight to twelve actively performed and monitored tasks per domain. These items are rated on one of two versions of three-point scales: (a) yes vs. incomplete/partially vs. no, or (b) very often/often vs. sometimes vs. rarely/never. The results are classified according to age-specific norm tables into “stanine values” (standard nine values). A stanine value of 1 corresponds to percentile ranks 0–4 and indicates an indicative finding. As these children solved fewer tasks successfully than 95% of children the same age, this result indicates a developmental risk. Further diagnosis by an external expert, for example a paediatrician, is recommended to parents or legal guardians by the preschool teachers. A stanine value of 2 corresponds to percentile ranks 5–11 and denotes an inconclusive finding. It is not possible to decide if the child is affected by a developmental risk at this point. In this case, further observation and a repetition of the DESK 3–6 R is recommended. Stanine values ranging between 3 and 9 correspond to percentile ranks 12–100 and indicate an age-appropriate development.

For 3- and 4-year olds the age specific norm tables are available in semi-annual steps to account for development in the age group (3 years and 0–5 months vs. 3 years and 6–11 months vs. 4 years and 0–5 months vs. 4 years and 6–11 months). Regarding 5- and 6-year olds there is one norm table per age (5 years vs. 6 years). The norm tables make sure that children do not simply grow out of a developmental risk. For example, if a 5-year-old child solves four tasks in the fine motor domain correctly they receive a stanine value of 3, which indicates no finding. If the same child solves the same number of tasks correctly one year later at the age of 6 they only receive a stanine value of 2, which is an indicative finding needing further attention.

Additionally, general information regarding the child and preschools is collected including the child’s age, how long the child has attended preschool, disabilities, additional developmental support outside the preschool such as speech therapy or physiotherapy, nationality, first language, and acceptance of the “circus-game”.

In addition to the DESK 3–6 R standardized questionnaires are delivered annually to the directors of the DESK preschools. These questionnaires serve to assess data specific to the individual preschool, e.g. the number of children, the participation in other funded programs, the contents of the targeted individual promotion, the number of additional weekly working hours, the cooperation with other DESK-preschools and with elementary schools. In 2020, additional questions were added regarding the COVID-19 pandemic and the implementation of preventive measures taken by the government.

## Results

Study 1: Effects of the targeted intervention on children affected by attentional and concentration developmental risks were examined longitudinally on basis of n = 940 children participating in SW1 and 2 using prevalence rate ratio (PRR) [12]. The DESK scores 1 and 2 were combined into one category “developmental risk/inconclusive finding” while scores 3–9 form the category “normal development”. The results show a significant decrease in the number of children affected by developmental risks.

Study 2: Another study published examined risks in the social development in n = 5,595 children [18]. The results show that 9.6% of all participating children yielded an indicative finding. For another 6.2% the findings were inconclusive. The prevalence of developmental risks varies between age groups and sex. There are indicative findings for 8.5% of 3-year olds (n = 119 out of 1,393) and inconclusive findings for another 6.4% (n = 89 out of 1,393). These two combined reveal that about 15% of 3-year olds are at risk for a developmental delay. Of the n = 1,673 4-year olds about 18% and 15% out of the n = 2,529 5- to 6-year olds were found to have developmental risks. For boys the prevalence of indicative and inconclusive findings was higher than for girls. While only about 10% of girls (n = 289 out of 2,810) showed inconclusive and indicative findings, over 21% of boys (n = 598 out of 2,785) were found to be at risk for developmental delays. Further risk factors are the presence of chronic diseases or disabilities, and indicative findings in the domains fine motor, gross motor and language/cognition [18].

Study 3: A total of 104 DESK preschools (response rate: 96.3%) responded to a semi-structured questionnaire assessing targeted individual promotion conducted by DESK preschools [9]. Although all DESK preschools receive additional financial resources, many tend to conduct rather unspecific measures of targeted individualized promotion which do not seem to differ much from activities already implemented in the preschools. Furthermore, the access of the pedagogic staff to evidence-based programs to promote children´s competencies seems to be rather limited.

Study 4: We also examined the effectiveness of the additional financial support the preschools receive [19]. Results reveal that additional staff hours are associated with a decrease in the prevalence of children with developmental risks. This is probably due to better care for each individual child if there are more teachers per child available to monitor and provide support. This has led to a recommendation addressed to the funding ministry that its funding should primarily be spent on additional working hours or additional staff.

Study 5: An ongoing discourse addresses a possible gender gap in achievements between boys and girls in education at preschool age. An analysis of gender differences was performed using data of n = 4,251 children screened with the DESK 3–6 comparing results conducted with gender-specific and gender-nonspecific norm tables [20]. The results using gender-nonspecific norm tables showed differences favouring girls in all age groups and domains (0.18≤|d|≤0.82). Contrarily, using gender-specific norm tables led to generally negligible gender differences and the few statistically significant differences were quantitatively rather small (0.005≤|d|≤0.42). This shows that competency-based, intersectional, individual-centred strategies capture the developmental status much better than solely focussing on the categorization of sex. The reason for not using sex-specific norm tables in the survey is simply that even though boys and girls at the same age are at different developmental stages, they still start school at the same age under the same conditions.

## Discussion

One of the main strengths of the cohort is the high-quality data of children in an important socio-spatial location. The DESK 3–6 R is a standardized, valid and reliable screening tool to catch developmental risks early [16]. Moreover, as it was developed together with preschool teachers it is therefore highly accepted in preschools [8]. The screening is conducted by preschool teachers who know the children well, but the measurement does not completely rely on their subjective evaluation. The reliability across all ages reaches Cronbach’s Alpha values ranging from α = 0.69 to α = 0.92 (for 3 year olds: 0.72 to 0.91; for 4 year olds; 0.71 to 0.88; 5 to 6 year olds: 0.69 to 0.92 [11]). Furthermore, the age-specific norm tables used are based on a sample of 1,693 children between the ages of 33 to 85 months. The validity of the scales for each domain has been assessed by correlating DESK scores with scores of other screening instruments, e.g. the Strengths and Difficulties Questionnaire (SDQ) [21].

Socioeconomic hotspots tend to present special challenges for early education. Longitudinally monitoring the development of children in these hotspots is an important step towards the development of evidence-based recommendations for both educational institutions such as preschools and elementary schools as well as lawmakers like the Ministry of Education and Daycare Facilities for Children Mecklenburg-Western Pomerania. Furthermore, the cohort provides trajectories on the basis of SW 1 to 3 (2017–2019) to compute expected values e.g. for SW 4 (2020) which can be compared with the measured values in SW 4. This method makes it possible to evaluate the effect of closing preschools as a preventive measure to stop the spread of COVID-19.

A limitation of the cohort refers to its generalizability because it is restricted to just one of the 16 German federal states. As mentioned in the introduction, compared to the German average, MWP differs from most other federal states due to its higher unemployment rate, lower household income, and a lower proportion of immigrants (2020: MWP 5.1% vs. Germany 13.7% [22]).

Another limitation is the lack of data about the quality of educational activities in individual preschools and the promotion of children’s competencies outside of preschools. Specifically, the cohort does not include indicators of the socioeconomic status of the parents like education levels or household income.

Furthermore, while the stanine values are age-specific the evaluation is not gender-specific despite the fact that gender-specific norm tables exist for each age group. This leads to a higher proportion of boys with a stanine value below 3. However, this should not be a problem in practice, because from the standpoint of wanting to prevent developmental risks it is ultimately better to declare more children at risk than overlooking children with a need for support.

The DESK 3–6 R itself has different limitations as a tool to assess cohort data. The DESK 3–6 and subsequently DESK 3–6 R were both conceptualized as tools to detect early signs of developmental risks which is why they are not optimized for cohort data analysis. A limitation is the inability to continuously compare all domains over all age groups (except for motor domains (Table 3)), which would be possible with instruments like the SDQ [21]. While instruments like the SDQ are continuous and could be used beyond preschool ages to allow for follow ups after starting school they are limited to the evaluation of specific competencies.

The DESK 3–6 R is a valid tool, which is easy to use for preschool teachers, and which captures a broad range of developmental domains. Compared to the use of an array of domain specific instruments preschool teachers need less training and, importantly, they can conduct the assessment in the setting of their daily work.

The value of the research lies in the early detection of developmental risks and being able to counter them before they may turn into developmental delays by intensifying promoting activities. As the results show there is evidence for the effectiveness of the targeted individual promotion to foster children’s competencies and counter developmental risks. Detecting and countering them this early is very beneficial for the children who without this screening could go unnoticed until the school entry examinations [23, 24]. Starting the screening at age three instead is a great advantage and important step in working towards educational equity as longer lasting early-prevention shows to be more effective.

More information about the cohort can be found on the website of the Institute for Community Medicine or by contacting the project leader Prof. Dr. Wolfgang Hoffmann (E-Mail: wolfgang.hoffmann@uni-greifswald.de). In order to be granted access to the data a specific research question must be presented. The request is then subject to evaluation by the project team. For details please also contact Prof. Dr. Hoffmann.

## Data Availability

All data generated or analysed during this study are included in this published article.

## List of Abbreviations

DESK 3-6 R: Dortmund Developmental Screening for Preschools–Revision (Dortmunder Entwicklungsscreening für den Kindergarten–Revision)
MWP: Mecklenburg-Western Pomerania
KiföG M-V: Federal state law for child day-care and preschools in MWP (Kindertagesförderungsgesetz Mecklenburg-Vorpommern)
ICM-VC: Institute for Community Medicine, Section Epidemiology of Health Care and Community Health
SW: Survey Wave
E-PIX: Enterprise Identifier Cross Referencing
SDQ: Strengths and Difficulties Questionnaire
VSE: Vanessa Sophie Ernst
MF: Marco Franze
AK: Anika Kästner
WH: Wolfgang Hoffmann
